# HDL cholesterol in females in the Framingham Heart Study is linked to a region of chromosome 2q

**DOI:** 10.1186/1471-2156-4-S1-S98

**Published:** 2003-12-31

**Authors:** Kari E North, Lisa J Martin, Tom Dyer, Anthony G Comuzzie, Jeff T Williams

**Affiliations:** 1 Department of Epidemiology, University of North Carolina, Chapel Hill, North Carolina, 27514 USA; 2 Center for Epidemiology and Biostatistics, Cincinnati Children's Hospital Medical Center, Cincinnati, Ohio, 45229 USA; 3 Department of Genetics, Southwest Foundation for Biomedical Research, San Antonio, Texas, 78245 USA

## Abstract

**Background:**

Despite strong evidence for a genetic component to variation in high-density lipoprotein cholesterol levels (HDL-C), specific polymorphisms associated with normal variation in HDL-C have not been identified. It is known, however, that HDL-C levels are influenced in complex ways by factors related to age and sex. In this paper, we examined the evidence for age- and sex-specific linkage of HDL-C in a longitudinal sample of participants from the Framingham Heart Study.

To determine if aging could influence our ability to detect linkage, we explored the evidence for linkage of HDL-C at three time points, *t*_1_, *t*_2_, and *t*_3_, spaced approximately 8 years apart and corresponding respectively to visits 11, 15, and 20 for the original cohort and 1, 2, and 4 for the offspring and spouses. Additionally, to examine the effects of sex on linkage at each time point, we estimated the heritability and genetic correlation of HDL-C, performed linkage analysis of HDL-C, tested for genotype-by-sex interaction at a QTL, and performed linkage analysis of HDL-C in males and females separately.

**Results and Conclusion:**

In women, we found evidence for a QTL on chromosome 2q influencing HDL-C variation. Although the QTL could be detected in the combined sample of males and females at the first time point, the linkage was not significant at subsequent time points.

## Background

Despite strong evidence for an additive genetic component to variation in high-density lipoprotein cholesterol (HDL-C) levels, specific polymorphisms associated with normal variation in HDL-C have not been identified. It is known, however, that HDL-C levels are influenced in complex ways by factors related to age and sex [1]. With specific reference to the Framingham Heart Study, Sonnenberg et al. [2] found that dietary fat and alcohol showed different relationships to HDL-C in pre- and post-menopausal women, and Wilson et al. [3] showed that HDL-C levels declined with advancing age and increasing obesity. In light of these indications of age and sex differences in HDL-C variability, we elected to examine the evidence for sex- and age-specific linkage of HDL-C in a longitudinal sample of participants from the Framingham Heart Study.

## Methods

### Data

The study design and methods of the Framingham Heart Study have been detailed elsewhere [4,5]. Beginning in 1948, 5209 subjects between the ages of 28 and 62 years were enrolled in the original cohort study and, starting in 1971, 5124 of their offspring and spouses were enrolled. Participants were invited to attend regular follow-up visits every 2 to 4 years, for the cohort and offspring groups, respectively.

We examined fasting HDL-C at time points *t*_1_, *t*_2_, and *t*_3_, spaced approximately 8 years apart and corresponding respectively to visits 11, 15, and 20 in the cohort and visits 1, 2, and 4 for the offspring and spouses. We chose the earliest observations to maximize our sample size, and considered only those individuals for whom complete data were available for age, sex, and cohort, various age-by-sex interactions, and cohort effects. Data were also cleaned for outliers (defined here as any observation greater than 4 standard deviations from the global mean) to reduce the effect of trait non-normality (kurtosis) on the analysis [6]. The kurtosis of the trimmed dataset for HDL-C is 0.3, 0.5, and 0.4, at *t*_1_, *t*_2_, and *t*_3_, respectively.

Our final sample size was 1562 participants, including in total 663 parent-offspring, 1273 sibling, 445 avuncular, which are aunt/uncle-niece/nephew pairs, and 717 pairs of first cousins. In all, the sample included information on nearly 3300 relative pairs.

### Analytic methods

Univariate quantitative genetic analysis was done to partition the phenotypic variance of HDL-C into its additive genetic and environmental variance components using maximum likelihood variance decomposition methods. The initial analysis screened for the following covariates: sex, age, age-by-sex interaction, cohort, body mass index (derived from height and weight), drinking status, and hypertensive status. Any covariates whose effects were significant at the *p *= 0.10 level in the initial analysis were retained in subsequent analyses, even if the significance levels decreased after inclusion of other covariates. After the initial covariate screening, maximum likelihood methods were used to estimate the effects of covariates and additive effects of genes.

Next, we generated genome-wide LOD scores for HDL-C at three time points, implemented in the program package SOLAR, using the methods detailed by Almasy and Blangero [7]. A pair-wise maximum likelihood-based procedure was used to estimate multi-point IBD probabilities. To permit multi-point analysis for QTL mapping, an extension of the technique of Fulker and colleagues [8] was employed.

The basic variance components approach can then be extended to a multivariate framework [9,10]. In the multivariate linkage model, the phenotype covariance is further decomposed to include the genetic correlation between traits due to additive genetic effects and the shared effects of the QTL, such that the covariation between two individuals for two traits is given by:



where *a *and *b *can be trait 1 or 2 and ρ_g _is the additive genetic correlation between the two traits. This approach has been implemented in SOLAR version 2.0.

We next tested for linkage to HDL-C using a variance-components linkage model extended to include genotype-by-sex interaction at a QTL [11-13]. The expected genetic covariance between a male and female relative pair *i*,*j *is defined as:

**σ**_g *ij *_= 2**φ**_*ij *_ρ_g *ij *_**σ**_gM _**σ**_gF _+ **π**_*ij *_**ρ**_q *ij *_**σ**_qM _**σ**_qF_,

where **φ**_*ij *_is the coefficient of kinship between the two individuals, ρ_g *ij *_is the additive genetic component of the correlation between the expressions of the trait in the two sexes, and σ_gM _and σ_gF _are the genetic standard deviations for males and females, respectively; **π**_*ij *_is the probability that individuals *i *and *j *are IBD at a quantitative trait locus tightly linked to a marker locus; ρ_q *ij*_is the marker-specific component of the trait correlation between the sexes; and σ_qM _and σ_qF _are, respectively, the marker-specific genetic standard deviations for males and females. Under the null hypothesis of no genotype-by-sex interaction at the QTL, the male and female marker-specific variances are equal (σ^2^_qM _= σ^2^_qF_).

Lastly, because a significant genotype-by-sex interaction was found, we generated genome-wide LOD scores for HDL-C at three time points in males and females separately in the program package SOLAR using the methods detailed by Almasy and Blangero [7].

## Results

At *t*_1_, *t*_2_, and *t*_3_, the mean ages of the sample members from the longitudinal sample were 38.3, 46.5, and 54.8 years, respectively. The residual heritability of HDL-C was estimated as 0.42 ± 0.05 across all three time points (sexes combined). Age, sex, and age-by-sex interactions were significant between time points, where as cohort effects were significant only at the last observation, *t*_3_. The variance explained by covariate effects was nearly constant, varying from 18% at *t*_1 _to 20% at *t*_3_. In preliminary analyses we also considered body mass index (derived from height and weight), drinking status, and hypertensive status as covariates (*n *= 1506; data not shown). The results were not significantly different from those presented below, and we did not interpret these analyses further.

The genetic correlations of HDL-C between time points, for males and females separately, are shown in Table 1. Measures were highly correlated across all time points, and the correlations for males and females were not significantly different.

**Figure 1:**
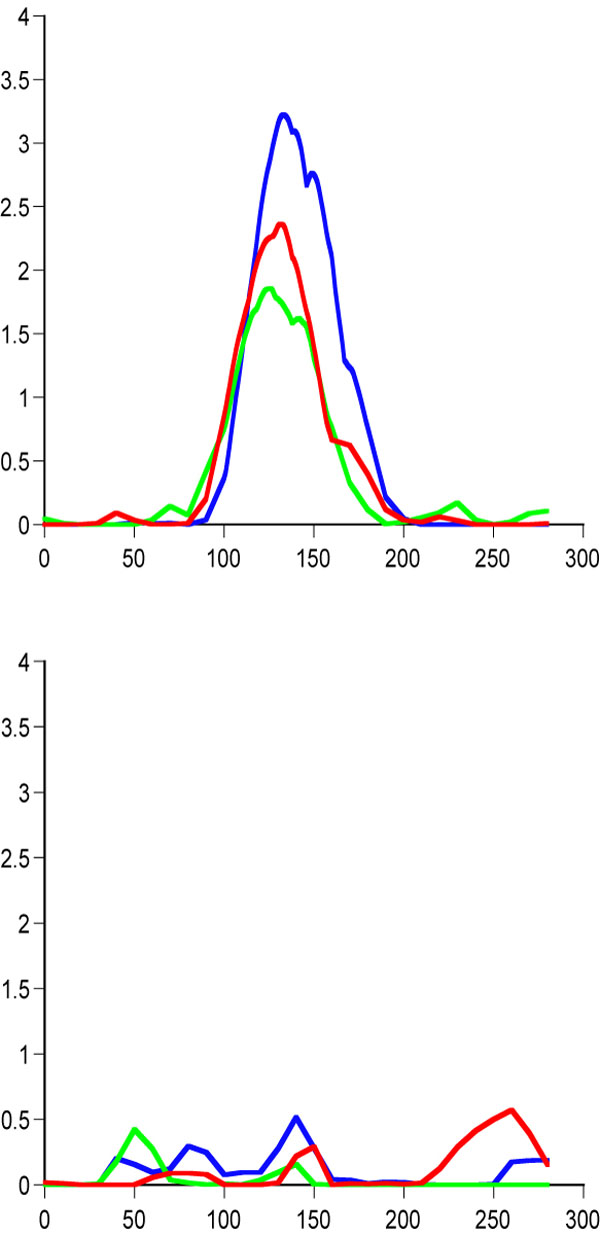
Genome-wide LOD score plots for HDL-C on chromosome 2 in females (left) and males (right) across all time points, *t*_1 _(blue), *t*_2 _(green), and *t*_3_

In a preliminary genome scan of HDL-C we obtained a maximum LOD score of 3.4 on chromosome 2 at 150 cM for the *t*_1 _observation. At *t*_2 _the maximum LOD score was 0.6 at 120 cM, and at *t*_3 _the maximum LOD was 1.1 at 122 cM. Based on these results we chose to restrict further analysis to chromosome 2 and to examine males and females separately at each time point. The results of separate linkage analyses in males and females are summarized in Figure 1 and Table 2 and suggest that, although the total additive genetic effect on HDL-C is not significantly different in males and females, sex does exert a strong effect on the QTL-specific variance of HDL-C.

We also considered the possibility of genotype-by-cohort and genotype-by-sex interactions on the HDL-C linkage, by formally modeling an interaction between sex and cohort in the linkage analysis (Table 3). All estimates of genotype-by-cohort interaction were nonsignificant (results not shown); at *t*_1_, however, males and females showed significantly different genetic variances (*p *= 0.01).

## Discussion

In a longitudinal sample of women from the Framingham Heart Study we found evidence for a QTL on chromosome 2q influencing HDL-C variation. Although the QTL could be detected in the combined sample of males and females at the first time point, the linkage was not significant at subsequent time points.

In sex-specific analyses, the QTL was detected consistently in females but not in males. Apart from the interaction with sex, our results are similar to those of Almasy et al. [14], who reported a LOD score of 2.3 at 140 cM on chromosome 2 for unesterified HDL-C.

Unfortunately, we did not have the desired covariate data to evaluate fully other possible sources of change across the time points, such as the influence of menopause, hormone therapy, oral contraceptive use, and nutrition. We therefore propose to examine further the evidence for sex-specific linkage of HDL-C in future studies of the Framingham Heart Study.

A search of genome databases revealed two plausible candidate genes located on chromosome 2q near marker *D2S1326*; these are the phospholipase A2 receptor 1 (PLA2R1) at 2q23–2q24 and the oxysterol binding protein-like 6 receptor (OSBPL6) at 2q32.1. Studies have demonstrated that estradiol affects PLA2R1 activity [15] and a relationship between secretory phospholipase A2 and HDL-C levels [16,17]. Oxysterol binding protein-like 6 is an intracellular lipid receptor that may have a regulatory role in the synthesis of cholesterol [18,19].

## Authors' contributions

KEN and LJM performed statistical analyses and interpreted results. JW assisted in the interpretation of the results. TD calculated the mIBDs. AGC participated in the design of the study. All authors read and approved the final manuscript.

## Figures and Tables

**Table 1:** Genetic correlation between HDL-C at three time points for males (upper triangle) and females (lower triangle).

	*t*_1_	*t*_2_	*t*_3_
*t*_1_	-	1.0	1.0
*t*_2_	0.96 ± 0.07	-	0.98 ± 0.06
*t*_3_	0.91 ± 0.07	0.88 ± 0.08	-

**Table 2:** Maximum LOD scores on chromosome 2 in separate linkage analyses of male and female subjects.

	Heritability^A^	Chromosome 2 Maximum LOD	Centimorgans (cM)
Females			
*t*_1_	0.48 ± 0.10	3.2200	133.0000
*t*_2_	0.42 ± 0.10	1.8600	126.0000
*t*_3_	0.38 ± 0.09	2.3600	132.0000
Males			
*t*_1_	0.62 ± 0.10	0.5200	140.0000
*t*_2_	0.50 ± 0.10	0.1600	140.0000
*t*_3_	0.51 ± 0.09	0.2900	150.0000

^A^All heritabilities significant at *p *= 0.0001.

**Table 3:** Genotype×sex linkage results from chromosome 2 at 135 cM.

	Mean	σ_g_	σ_e_	σ_q_	*h*^2^_g_	*h*^2^_q_
Females						
*t*_1_	58.38 ± 0.89	2.20 ± 2.19	10.16 ± 0.73	9.75 ± 0.93^A^	0.0200	0.4700
*t*_2_	57.70 ± 0.82	5.96 ± 1.86	9.50 ± 0.77	6.51 ± 1.63	0.2100	0.2500
*t*_3_	52.48 ± 0.87	5.86 ± 1.81	10.19 ± 0.77	6.94 ± 1.61	0.1800	0.2600
Males						
*t*_1_	44.64 ± 1.37	7.66 ± 1.25	7.25 ± 0.88	4.25 ± 1.48^A^	0.4500	0.1400
*t*_2_	45.87 ± 1.28	7.56 ± 0.93	7.34 ± 0.72	1.11 ± 2.22	0.5100	0.0100
*t*_3_	39.69 ± 1.51	7.78 ± 1.16	8.19 ± 0.70	2.56 ± 2.21	0.4500	0.0500

^A^At *t*_1 _σ_q,F _≠ σ_q,M _*p *= 0.01.
